# Dynamics of astrocytes Ca^2+^ signaling: a low-cost fluorescence customized system for 2D cultures

**DOI:** 10.3389/fcell.2024.1320672

**Published:** 2024-01-23

**Authors:** Rosa Musotto, Ulderico Wanderlingh, Angela D’Ascola, Michela Spatuzza, Maria Vincenza Catania, Maurizio De Pittà, Giovanni Pioggia

**Affiliations:** ^1^ Institute for Biomedical Research and Innovation, National Research Council (IRIB-CNR), Messina, Italy; ^2^ Department of Mathematical and Computer Sciences, Physical Sciences and Earth Sciences, University of Messina, Messina, Italy; ^3^ Department of Clinical and Experimental Medicine, University of Messina, Policlinico Universitario, Messina, Italy; ^4^ Institute for Biomedical Research and Innovation, National Research Council (IRIB-CNR), Catania, Italy; ^5^ Division of Clinical and Computational Neurosciences, Krembil Research Institute, University Health Network, Toronto, ON, Canada; ^6^ Department of Physiology, Temerty Faculty of Medicine, University of Toronto, Toronto, ON, Canada; ^7^ Basque Center for Applied Mathematics, Bilbao, Spain; ^8^ Department of Neurosciences, Faculty of Medicine, The University of the Basque Country (UPV/EHU), Leioa, Spain

**Keywords:** astrocytes, calcium waves, fluorescence, customized system, analysis

## Abstract

In an effort to help reduce the costs of fluorescence microscopy and expand the use of this valuable technique, we developed a low-cost platform capable of visualising and analysing the spatio-temporal dynamics of intracellular Ca^2+^ signalling in astrocytes. The created platform, consisting of a specially adapted fluorescence microscope and a data analysis procedure performed with Imagej Fiji software and custom scripts, allowed us to detect relative changes of intracellular Ca^2+^ ions in astrocytes. To demonstrate the usefulness of the workflow, we applied the methodology to several *in vitro* astrocyte preparations, specifically immortalised human astrocyte cells and wild-type mouse cells. To demonstrate the reliability of the procedure, analyses were conducted by stimulating astrocyte activity with the agonist dihydroxyphenylglycine (DHPG), alone or in the presence of the antagonist 2-methyl-6-phenylethyl-pyridine (MPEP).

## 1 Introduction

Live cell imaging plays a central role in biological and medical research, enabling scientists to explore and understand cellular processes in intact organisms in a dynamic and detailed manner. Imaging techniques provide valuable information on the morphology, function, dynamics and interactions of cells under physiological conditions. The use of microscopes capable of capturing the dynamics of increasingly rapid biological events, fluorescent probes, fluorescent proteins, and other labeling technologies, together with data reconstruction and analysis programs, make it possible to study the localization and dynamics of specific molecules within cells. However, when large spatial and temporal resolutions are required, live imaging is still difficult, as spatial resolution increases at the expense of temporal resolution. Furthermore, advanced imaging tools, such as confocal microscopes, fluorescence microscopes, and real-time cell monitoring equipment, can have a significant initial cost. This paper presents a method that combines a fluorescent light probe, a specially adapted microscope involving the use of digital cameras, and a customized post-processing technique that allows rapid dynamic processes within cells to be observed in real-time. In particular, the proposed approach has the advantage of achieving, at low cost, a good compromise between temporal resolution (i.e., frame rate) and spatial resolution. The optimal settings of fluorophore concentration, illumination time and intensity were determined in order to achieve the detection of calcium dynamics inside live cells. The adapted fluorescence microscope produces images that are comparable to those of a confocal microscope but has much faster acquisition times, which made it possible to detect even “calcium transients” that occur within seconds or fractions of seconds.

For many years, it was believed that only neurons could produce and control brain signaling, while the glia surrounding them only served as structural and metabolic support for neuronal function. Recent physiological studies have radically changed this view, suggesting that glial cells, and in particular astrocytes, not only play a supporting role in neuronal function, but are also engaged in communication, with each other and with neurons by dynamically interacting with synapses through the uptake and release of gliotransmitters mediated by intracellular Ca^2+^ signaling receptors (Volterra and Meldolesi, 2005; Haydon and Carmignoto, 2006b; Clarke and Barres, 2013; Khakh and McCarthy, 2015; Verkhratsky and Nedergaard, 2018; Semyanov et al., 2020; Kofuji and Araque, 2021).

Intracellular Ca^2+^ signaling is crucial in many cellular processes, such as muscle contraction, neurotransmitter release and gene expression (Berridge et al., 2000; Clapham, 2007). The fundamental and varied roles that calcium plays in brain development are numerous. Ca^2+^ has been demonstrated to control a variety of processes involved in learning and memory, including synaptogenesis, synaptic transmission, energy production, membrane excitability, and neuronal gene expression (Basarsky et al., 1994; Finkbeiner and Greenberg, 1998; Bootman et al., 2001; Berridge et al., 2003; Haydon and Carmignoto, 2006a). Given the central role that Ca^2+^ plays in brain physiology, it is not surprising that even small changes in Ca^2+^ homeostasis result in significant functional changes. Deregulation of Ca^2+^ homeostasis and its signaling is a hallmark of both age-related diseases and neurodegenerative disorders (Gleichmann and Mattson, 2011; Zündorf and Reiser, 2011; Schrank et al., 2020). However, the precise spatio-temporal oscillations of calcium in cells are very complex and dynamic. Therefore, understanding the spatio-temporal dynamics of calcium in cells is essential for revealing the mechanisms of many biological processes and to develop new therapies for diseases.

Recent advances in microscopy and imaging techniques have provided new tools to study the spatio-temporal dynamics of calcium in living cells with high spatial and time resolution. This field of research, known as calcium imaging, has opened new avenues for understanding the complexities of calcium signaling and its role in cellular physiology and pathology (Mekahli et al., 2011; Giorgi et al., 2018a).

Intracellular and intercellular calcium waves play a key role in cellular communication between astrocytes and between astrocytes and neurons and it as been shown that astrocytes, although electrically silent, actively participate in synapses through calcium modulation (Pasti et al., 1997; Carmignoto, 2000). Astrocytes respond to external stimulation due to neuronal activity through increases in intracellular Ca^2+^. Calcium waves (ICW) are also able to cross the cell membrane and stimulate adjacent astrocytes through gap junctions (Cornell-Bell et al., 1990; Volterra et al., 2014; Armbruster et al., 2022). Beyond gap junctions calcium waves can also propagated through extracellular messengers located in the extracellular space such as ATP and glutamate (Boitano et al., 1992; Paemeleire and Leybaert, 2000; Rouach et al., 2000; Nedergaard et al., 2003; Scemes and Giaume, 2006; Leybaert and Sanderson, 2012).

The coupling of glutamate to specific receptors located on the cell membrane of astrocytes triggers a chain of events that leads to the production of IP_3_ (inositol 1, 4, 5-triphosphate) and the release of gliotransmitters (Kofuji and Araque, 2021). More specifically, glutamate, which is the neurotransmitter most abundant in the brain, binds to its membrane receptor located on the membrane of astrocytes, interacts with the G protein and forms a receptor-G protein complex on the inner surface of the membrane which leads, after a series of reactions, to the production of Inositol trisphosphate IP_3_. The formed IP_3_ molecules diffuse into the cytosol and bind to a specific receptor for IP_3_ located on the surface of the Endoplasmic Reticulum (ER). Binding to IP_3_ opens the IP_3_ receptor-channel, causing Ca^2+^ ions to be released from the ER-store into the cytoplasm which, in turn, induces the opening of ryanodine receptors in the ER, resulting in the further release of Ca^2+^ into the cytosol. This phenomenon is called calcium-induced calcium release (CIRC) (LARTER and CRAIG, 2005).

The spatio-temporal dynamics of Ca^2+^ waves in astrocytes have been identified (Sternberg Stanley, 1983a) through the development of new imaging technologies and the use of fluorescent indicators capable of specifically marking the cellular components of interest.

In this article, we adopt a method for monitoring and analyzing changes in intracellular Ca^2+^ concentration in real time using an adapted epifluorescence microscope and a low molecular weight fluorescent calcium indicator, Fluo-8. The experimentally observed intracellular Ca^2+^ waves were analyzed using a specialized ImageJ cell analysis software in combination with customized scripts in order to describe the spatio-temporal mechanisms underlying ion diffusion and the calcium-dependent correlation in communication between astrocytic cells.

## 2 Materials and methods

### 2.1 Astrocytic cultures

Cultures of immortalized cortical astrocytes of human origin were provided by Innoprot (Spain). Cells were cultured in 25 cm^2^ flasks treated with poly-L-lysine in 5 mL of astrocyte medium with the addition of 2% fetal bovine serum (FBS), astrocyte growth supplements (AGS) and an antibiotic solution containing penicillin (100 U/mL) and streptomycin (100 µg/mL), at 37°C in humidified air with 5% CO_2_. All cell culture reagents are supplied by Innoprot (Spain).

Primary astroglial cultures were isolated from the cerebral cortex of newborn (post-natal day P0-P1) wild type (WT) mice of the mouse strain C57BL6/J. Animals used to obtain cultures came from a resident colony in the animal facility of the University of Catania according to the Directive of the European Communities Council (2010/63/EU) for animal use in Neuroscience Research (Project #379—approval number 556/2022—PR). Coltures were prepared as described in Di Marco et al. (DI MARCO et al., 2021), with minor modifications: cells were plated on T75 flasks (one cortex per flask, about 5.000.000 cells/pup) in Dulbecco’s modified Eagle medium (DMEM) containing glucose (4500 mg/L), L-glutamine (584 mg/L), sodium-pyruvate (1 mM), 10% FBS (fetal bovine serum) and an antibiotic solution containing penicillin (100 U/mL) and streptomycin (100 µg/mL). When cultures reached 90% confluence, the cells were trypsinized, counted, and reseeded at a density of 4 × 10^4^ cells/cm^2^ in T25 flasks. In the next passage, after trypsinization, cells were counted and plated at the density of 5 × 10^4^/well in 24-well plates for treatments.

### 2.2 Cell treatments

The astrocytes were seeded in 24-well plates, in order to reach approximately 90% confluence at 24 h. The culture medium was replaced by 0.4 mL of fresh 0.5% FBS medium (with the exception of the FBS, the composition of the culture medium was the same as that used for the growth of the cell culture); and 5 μM of Fluo-8 AM, a calcium-binding fluorescent green dye (Abcam, United States) was added. After 45 min in the incubator, the plates were placed in a laminar flow hood to perform the treatments. The cells were washed, and the medium was replaced with Dulbecco’s phosphate-buffered saline (DPBS) (37°C), a balanced saline solution containing Ca^2+^, Mg^2+^ and glucose 15 min prior to the addition of 10 μM metabotropic group I glutamate receptor agonist 3,dihydroxyphenylglycine (DHPG) (Tocris). A further set of plates were treated with a potent and highly selective mGlu5 receptor inhibitor, 2-Methyl-6-(phenylethinyl) pyridine (MPEP) (Tocris) at a concentration of 50 μM, 15 min before adding the agonist DHPG to allow a complete receptor blockade. After exposure to drugs, the plates were rapidly put on the specimen stage of our optical system located in the incubator.

### 2.3 Optical system

A key method in biomedical research is microscopy. Time-lapse imaging is particularly helpful since it enables the study of cell dynamics both *in vitro* and *in vivo* (Coutu and Schroeder, 2013). The requirement for strict environmental control to ensure normal cell behavior during the imaging time is one of the primary causes for the high cost of live imaging systems. As a result, more expensive equipment is needed to maintain a constant and ideal temperature and pH conditions for cell growth, to reduce phototoxicity by limiting light exposure, and to prevent variations in osmolarity by reducing evaporation.

**FIGURE 1:**
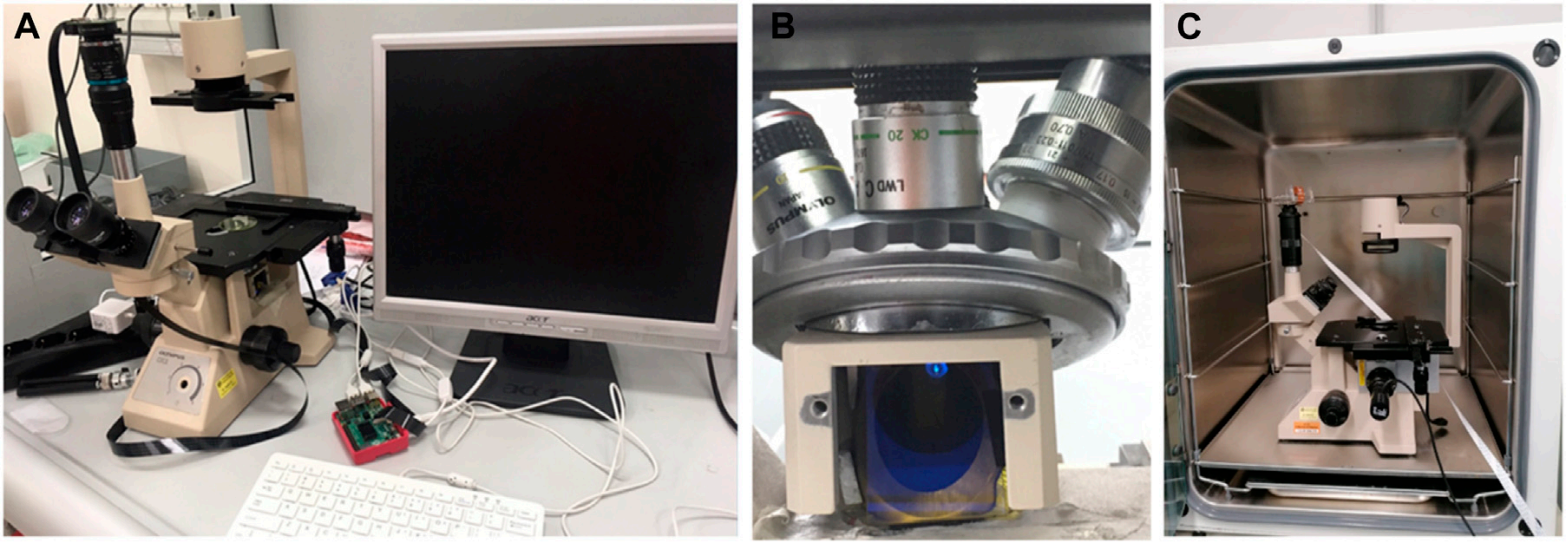
Specially adapted inverted fluorescence microscope **(A)** Picture of the adapted microscope and its supplements. **(B)** Detail of the positioning of the dichroic mirror **(C)** Housing of the adapted microscope inside the incubator.

Since it was necessary to observe the Ca^2+^ signal in astrocyte cell cultures under controlled conditions close to the physiological ones, i.e., T = 35°C, CO_2_ saturation and 77% humidity, an inverted microscope was modified for tissue and cell cultures, an Olympus CK2, in order to place it inside an incubator for the cell cultures, a Galaxy S plus (RS Biotech Laboratory Equipment Ltd, United Kingdom) i.e., where an ordinary fluorescence microscope could not be located. The used inverted microscope of about 20 cm × 20 cm × 30 cm was fitted in a standard 50 lt incubator (see Figure 1).

The original inverted microscope was modified by inserting a 45° dichroic mirror (Alluxa 505 Ultra Longpass) in the optical path from the objective to the eyepiece, laterally illuminated by a high-luminosity LED (Thorlab M470L4), equipped with a filter (Alluxa 483.5-25 OD6 Ultra). A further filter (Alluxa 524-32 OD6 Ultra) was placed near the eyepiece to filter out the excitatory wavelength at 488 nm and let through only the fluorescence wavelength. The second filter was housed in the existing structure of the microscope near the eyepiece.

The system for direct illumination of the sample was also modified by replacing the original incandescent lamp of the optical condenser with a white LED light whose switching and brightness is controlled via a Raspberry computer, thus also making it possible to obtain bright-field images of the sample. The selection parameter for the LED to be used was that of maximum light intensity transmission through the excitation filter window.

In the availability of LED sources on the market, the choice fell on Thorlabs’ M470L4, which, although not well centred in the transmission window, manages to transfer more energy than the M490L4 due to its higher irradiance.

The images were recorded using high-resolution CMOS sensors, adapted to the microscope optics and inter-faced with a Raspberry PI4 computer to automate the acquisition sequences. The Raspberry Pi4 model B is a mini PC equipped with a quad-core 1.5 GHz 64-bit ARM Cortex-A72 CPU, with a dedicated camera GPU, 2 micro-HDMI, 4 GB RAM, with LAN access, WiFi, USB 3.0 and bluethoot.

The system disk is installed on a microSD card and is able to offer pre-stations comparable to entry-level x86 PC systems.

On the microSD it is possible to install various operating systems of the Linux family, among them Raspbian based on the Linux distribution Debian. Finally, it is equipped with a standard 40-pin GPIO general-purpose input/output connector, which allows connecting, configuring and managing electronic/digital devices. Two different sensors were tested in image recording: IMX477 (Native-camera, 2023) and IMX462 (Pivariety-Camera, 2023) both manufactured by Sony and available in the High Quality Camera by Raspberry inc. and the Ultra Low Light Camera by Ar- ducam. The first sensor has a resolution of 12Mpixel, while the second has a lower resolution (2Mpixel) and a higher sensitivity. sensitivity. While the first sensor allows the acquisition of excellent images with high detail, it was less effective in the acquisition of time-lapse sequences. In fact, the phenomenon of photo-bleaching of the fluorophore was significant after a few acquisition sequences.

In contrast, using the IMX462 sensor it was possible to reduce the illumination on the sample and minimise the photo-bleaching phenomenon. The integrated use of the devices used and connected with the Raspberry was implemented in the Python language using the various available libraries. In particular, the use of the libraries PiCamera and Libcamera, aimed at supporting the cameras directly from the Linux operating systems, allowed us to have easy access to the different parameters for the acquisition of images, such as framerate, sensitivity, speed of the camera and so on. This is particularly important in the acquisition of technical images where the parameters must be the same during the registration.

**FIGURE 2:**
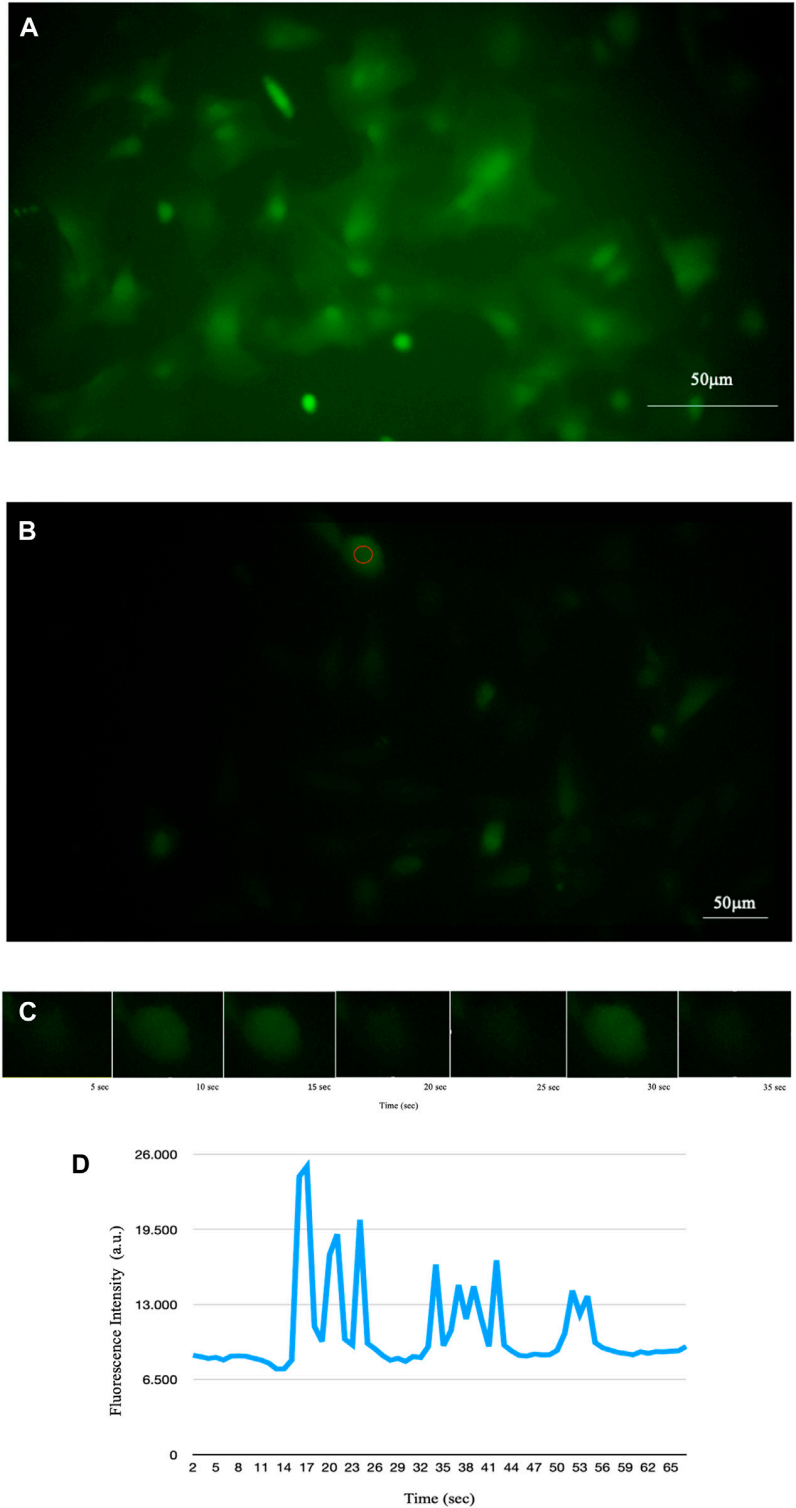
Fluorescence of intracellular Ca^2+^ signal of astrocytes detected by time-lapse methodology. **(A)** Fluorescence of a sample of mouse astroglial cells detected in time-lapse of one frame every 5 s (PCLWDCD20XPL NA 0,40 W.D.5,4 mm). **(B)** Fluorescence of a sample of human astrocyte cells detected in time-lapse of one frame every 5 s (PCD10XPL NA 0,25 W.D. 7,2 mm). **(C)** Example of some time-lapse frames of the cell circled in B. Using time-lapse technology, it is possible to observe the increase in intracellular calcium and its diffusion through astrocytes in real time. **(D)** Representative trace of Ca^2+^ in the cell underlined in red in B showing temporal changes in the signal.

We then developed several codes that acquire a bright-field image and then a series of time lapse exposures, synchronised with the switching on of the excitation LED (see Figure 2).

With similar codes, fast acquisitions were also realised, in particular with the IMX462 sensor, capturing up to 200 frames per second.

### 2.4 Description of the analysis workflow

We first identified regions of interest (ROIs) by manually applying a circle of a known area to each astrocytic cell. We then extracted the fluorescence intensity. Next, we identified “peaks”, which were used to calculate the summary and aggregate metrics per image and per cell to assess network and cell activity.

#### 2.4.1 Step 1: opening the sequence of images collected by the camera

For each imaging field, a time series of images was collected and saved using a file.tif format to maintain image metadata. The intensity changes, detected by the fluorescence microscope, were processed with the ImageJ with the Fiji distribution for biological image analysis. ImageJ uses modern engineering practices to combine powerful software libraries with a wide range of scripting languages to enable rapid prototyping of image processing algorithms (Johannes et al., 2012; Schneider et al., 2012).

#### 2.4.2 Step 2: correction of an unevenly illuminated background

An algorithm called the “Rolling ball radius” was used, which works like a filter. Let us imagine that we immerse a ball below the surface at the desired location. Once it is completely covered by the blocks, the apex of the ball determines the intensity of the background at that position. By rolling the ball under the surface, the background values for the entire image are obtained. The radius of the sphere was set to a value greater than the largest astrocyte structure (Sternberg, 1983b). The option “Sliding paraboloid” was used: here the rotating sphere is replaced by a sliding rotating paraboloid with the same curvature at the vertex as the sphere of the given radius. The paraboloid has the advantage that suitable paraboloids can be found for any image value, even if the pixel values are much larger (in pixels) than the typical object cell size.

**FIGURE 3:**
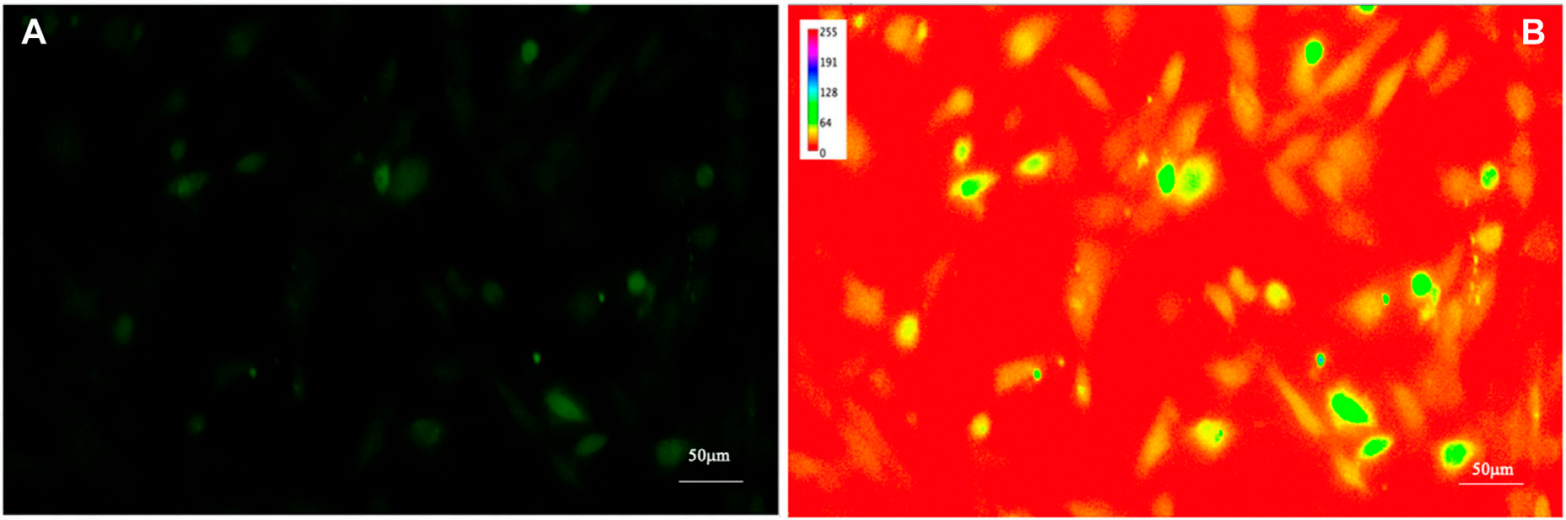
Fluorescence image of intracellular Ca^2+^ of human astrocytes. To increase the visibility of low-contrast features **(A)** and help the human eye to compare different images by enhancing the differences in intensity of the samples, a look-up table was applied **(B)**.

To increase the visibility of low-contrast features and help the human eye to compare different images, by enhancing the differences in intensity of the samples, a spectrum look-up table was applied (see Figure 3); the spectrum look-up table maps intensities to the standard color ROYGBIV spectrum from red (low values) to violet (high values).

#### 2.4.3 Step 3: identify ROIs

**FIGURE 4:**
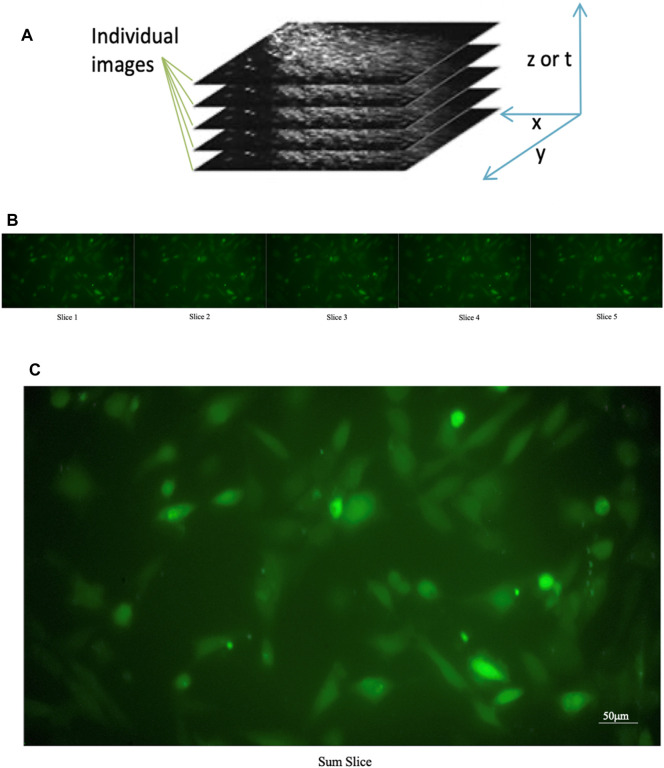
Sum of the individual images of a time series. **(A)** Construction of a “slice sum”; all pixels with the same xy coordinates are summed up. The time dimension is used as the z-direction **(B)** Original individual stack **(C)** Sum of the individual images of a time series.

A single image consisting of the sum of temporally correlated images (see Figure 4) superimposed on each other was produced. Using the “Slice Sum” function, it is possible to sum the pixels in the z-direction. Since films do not have a z-direction, the time dimension is used as the z-direction. Then all pixels with the same xy coordinates are summed up. Each pixel value affects the result, which is an advantage if you want to measure intensities in the projection image. On the resulting image, we affix ROIs, so that we are sure to have an ROI on each cell. Subsequently, all ROIs are transposed onto the film to enable the analysis of individual cells.

To determine the best position of the ROI within the astrocyte cells, the results of different ROI selections on the same astrocyte were compared. On the Stack image created, a circular ROI was marked in the center of each cell with the “*circle*” tool (see Figure 4). Each ROI was named and added to the ROI Manager tool.

#### 2.4.4 Step 4: intensity measurement

**FIGURE 5:**
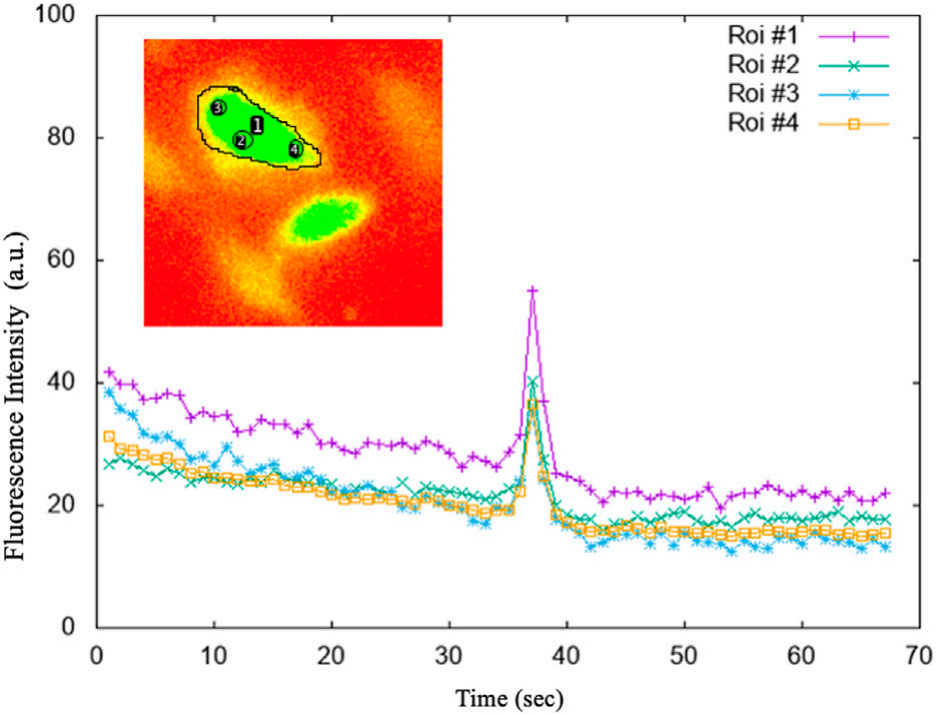
The average intensities of the selected regions of interest (ROI). ROI#1: drawn on the entire astrocytic cell; ROI#3 and ROI#4 on the sides of the astrocytic cell; ROI#2 in the centre of the astrocytic cell. The graphs of the fluorescence versus time of the individual 4 ROIs, shown in the figure, highlighted that whatever is measured within the astrocytic cell, the Ca^2+^ signal is both evident and comparable.

The ROIs performed on the stack image were transposed onto the time sequence of images. From each identified astrocyte, i.e., ROI, we extracted calcium signals by measuring the fluorescence intensity over time. With the help of a customized script on Gnuplot we extracted the graphs and the corresponding data file of the fluorescence intensity as a function of time for each individual cell. A second customized script on Gnuplot made it possible to determine the intracellular Ca^2+^ peaks of each astrocyte by calculating Topographic Prominence (TP) (Llobera, 2001), which measures the vertical distance between the peak and its lowest contour line. TP originated from the fields of geography and geology, and researchers in these fields use TP as a measure of the relative heights of peaks within their surroundings (see Figure 5). An analogy is possible between a mountainous landscape and the graph of the intracellular Ca^2+^ peaks of astrocytic cells.

**FIGURE 6:**
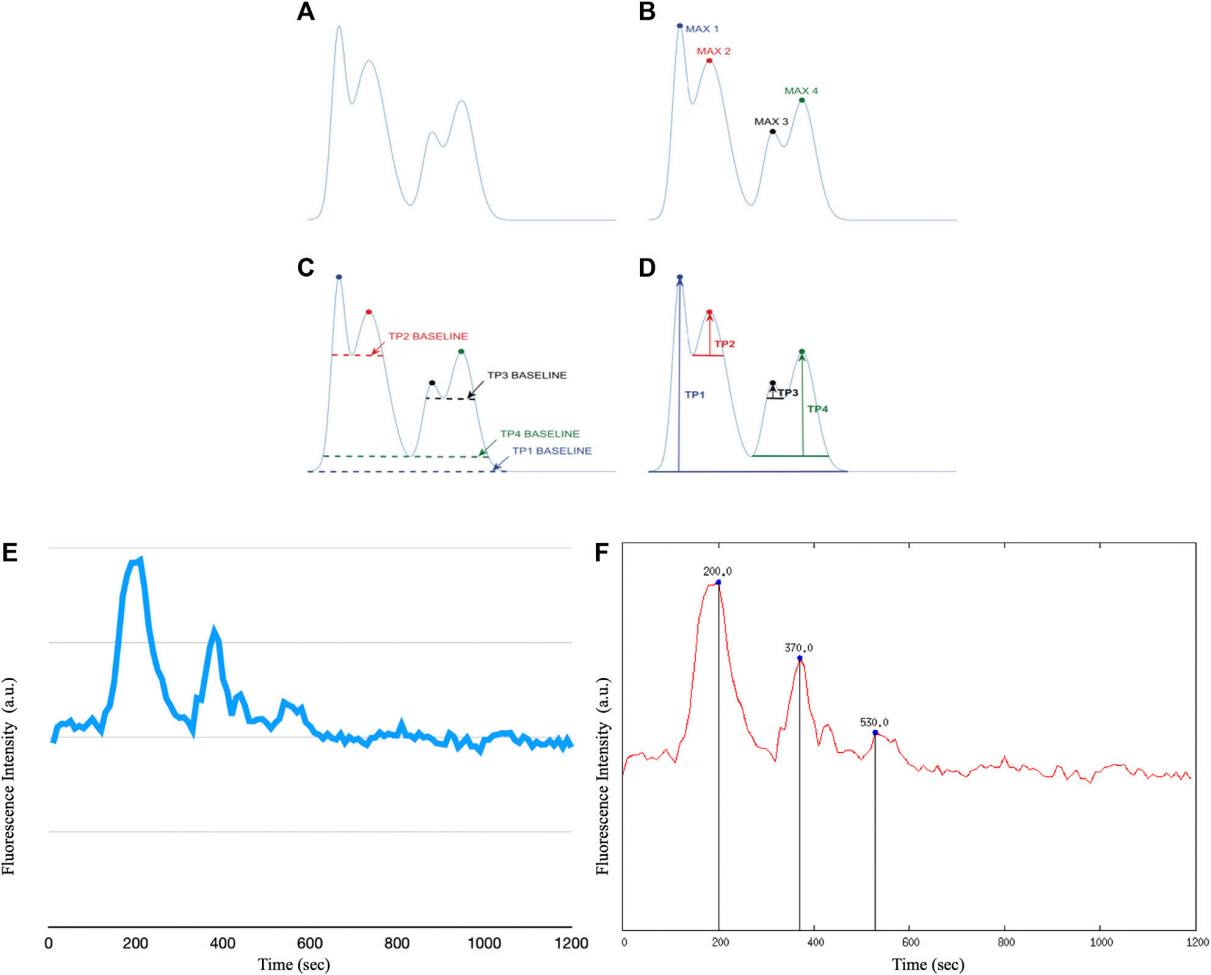
The TP calculation algorithm. We apply the TP method to the raw fluorescence intensity data of intracellular Ca^2+^ waves of astrocyte cells, however it is possible to apply the method to any type of raw data. **(A)** Example of a curve on which to apply the TP algorithm **(B)** Identification of the maximum points of the distribution. **(C)** Calculation of the lowest contour that encircles each maximum but not any higher peaks. **(D)** Calculation of TP as the relative height of each maximum above each baseline. **(E)** Example of raw recording of a Ca^2+^ signal in immortalized human astrocytes **(F)** Example of Ca^2+^ peak determination in immortalized human astrocytes after application of the Topographic Prominence algorithm.

Figure 6 shows all the steps of the post-processing technique. The customized scripts allowed the calculation of Ca^2+^ transients within each cell.

**FIGURE 7:**
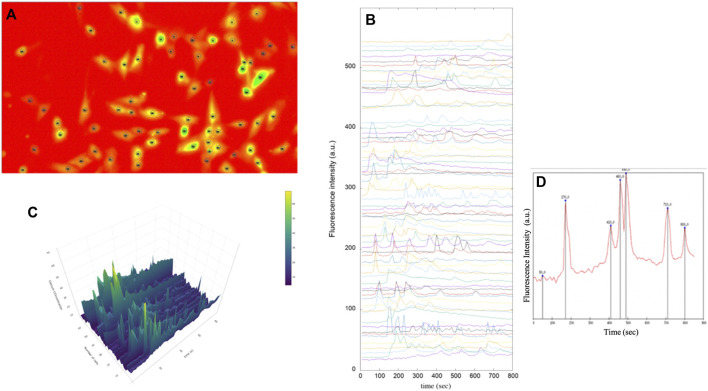
Fluorescence images of immortalised human astrocytes labelled with Fluo-8. **(A)** Identification of astrocytic cells and tracing of regions of interest (ROI). **(B)** Representative fluorescence intensity profiles showing spontaneous intracellular Ca^2+^ activity in immortalised human astrocytes under basal conditions. **(C)** 3D analysis of intensity intracellular Ca^2+^ in astrocytes **(D)** Intracellular Ca^2+^ peaks determined by prominence calculation by means of a Gnuplot script.

Figure 7 shows all the steps of the post-processing technique. The customized scripts allowed the calculation of Ca^2+^ transients within each cell.

## 3 Results

To demonstrate the usefulness of the workflow, we applied the methodology to different astrocyte preparations *in vitro*, specifically, immortalized human astrocytic cells and wild-type (WT) mouse primary astrocytic cells cultures.

**FIGURE 8:**
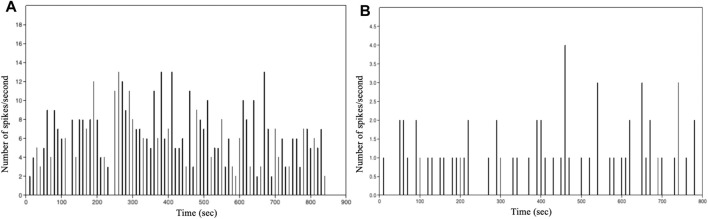
Histogram showing the number of spikes per second vs. time of intracellular Ca^2+^ events in: **(A)** immortalized human astrocytes under basal conditions. **(B)** WT mouse astrocytes under basal conditions.

The graphs in Figure 8 show the number of events per second vs. time of intracellular of Ca^2+^ events as a function of time for the analysed astrocytic cell types. This distribution enabled us to obtain valuable information on the astrocytes network dynamics. A first analysis shows that, with the same number of cells, the spikes per seconds of Ca^2+^ events varies depending on the analysed species.

To show that the procedure is reliable, analyses were conducted under basal condition and after stimulation of astrocyte activity.

**FIGURE 9:**
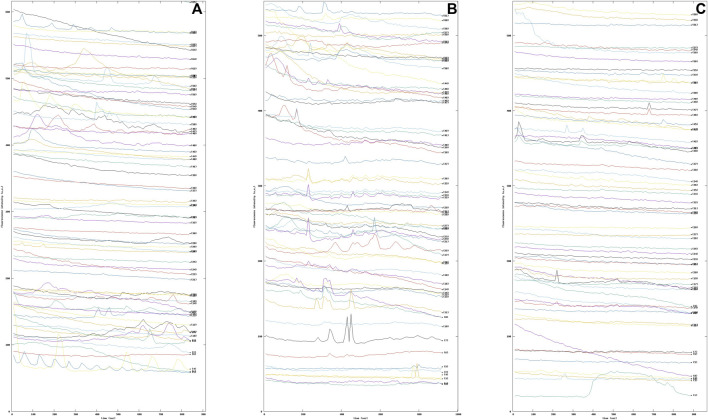
Sequence of fluorescence intensity profiles of intracellular Ca^2+^ in Wild Type (WT) mouse astrocytes **(A)** WT mouse astrocytes under basal condition **(B)** WT mouse astrocytes treated with 10 μM the selective metabotropic glutamate receptor agonist 3,5-dihydroxyphenylglycine (DHPG) mGluR5. **(C)** WT mouse astrocytes were pre-treated with 50 μM 2-methyl-6-(phenylethynyl)pyridine (MPEP), a selective antagonist of the metabotropic glutamate receptor subtype mGluR5, for 15 min and then exposed to the DHPG agonist.

The increase of intracellular Ca^2+^ in astrocytes during synaptic activity appears to be mainly attributable to activation of metabotropic glutamate receptor subtype 5 (mGluR5) (D’Antoni et al., 2008). In fact, it has been established that the glutamate-sensitive surface receptor mGluR5 regulates multiple forms of astrocyte-neuron interaction, including modulation of synaptic excitability and glutamate transport, particularly in early development (Romano et al., 1995). To this end, astrocytic cells were treated with the agonist dihydroxyphenylglycine (DHPG), alone or in the presence of the selective negative allosteric modulator of mGluR5, 2-methyl-6-(phenylethynyl) pyridine (MPEP). Again, our methodology was able to highlight the Ca^2+^ dynamics modified by the pharmacological activation of the mGlu5 receptors. Indeed, it can be seen from the graphs that the intracellular Ca^2+^ dynamics varied in the three samples examined (see Figure 9).

## 4 Discussion

Imaging of live samples in time-lapse is more challenging than imaging of fixed samples. The multidisciplinary approach and careful planning of the entire imaging protocol led to the detection and study of fast calcium waves within astrocytic cells. However, in general, the adapted epifluorescence microscope enables morphogenesis and cell dynamics to be studied with high temporal resolution. Time and spatial resolution cannot be considered independently; for example, laser scanning confocal microscopy (CLSM), probably one of the most widely used methods for *in vivo* imaging, has a very high spatial resolution but acquisition times that can be prohibitive. This technique, which is well suited for imaging stationary samples, cannot be used to study the fast processes of live cells (VERMOT et al., 2008). Dynamic processes in cell biology cover a wide range of time and space scales. Fast calcium waves, for example, have a speed of 10–50 μm∕sec (Jaffe and Créton, 1998). The adapted epifluorescence microscope in conjunction with the experimental determination of fluorophore concentration, time and illumination intensity produced images comparable to those of a confocal microscope and enabled all astrocytic cells to be viewed simultaneously for the study of network dynamics.

## 5 Conclusion

Due to nearly a century of primarily neuron-focused research, we know very little about the physiology of astrocytes. Astrocytes became a hot topic in neuroscience after it was discovered that they dynamically modulate synaptic functions and that they are involved in the early stages of neurological disorders such as epilepsy, ischemia, Alzheimer’s, and Parkinson’s diseases (Barres, 2008; Allaman et al., 2011; Pekny et al., 2016). The ability of astrocytes to release gliotransmitters following Ca^2+^ fluctuations confirms that these cells actively participate in information processing in the brain. The imaging methodology adopted in conjunction with the use of a next-generation fluorescent marker, Fluo-8, made it possible to detect the space-time dynamics of Ca^2+^ waves in human and WT mouse astrocytes.

This study clearly demonstrates that the employed method and the instrument especially adapted for the purpose, respond positively to the detection and subsequent evaluation of relative changes in intracellular Ca^2+^ ions in astrocytic cells. Furthermore, the low cost required to set up the microscope makes this method easily useable by any research laboratory. Cortical astrocytes were used as a model for this work; however, the developed survey methodology can be used to measure intracellular Ca^2+^ dynamics in a variety of cells in real time.

### 5.1 Limitations and future work

Although the created method responds effectively to the detection and subsequent evaluation of relative changes in intracellular Ca^2+^ ions in astrocytic cells, it has limitations due to the time required for ROI selection. On the other hand, existing methods for automatic detection of ROIs fail to achieve sufficient accuracy and reliability. Furthermore, although the microscope adapted in conjunction with the proposed method manages to produce images comparable to those of a confocal microscope, the latter offers greater depth resolution resulting in better spatial and temporal resolution than the approach we tested (WANG et al., 2017). It should also be emphasised that the approach used is only suitable for monolayer preparations, whereas confocal microscopy is particularly suitable for acquiring three-dimensional images at the level of individual thin sections of the sample. Future work, in addition to the realization of a separate and reliable ROI detection method, will be aimed at the detection and evaluation of the intracellular calcium signal in astrocytic cells from mice with neurodegenerative disorders in order to compare the network dynamics between healthy and diseased mice.

## Data Availability

The datasets presented in this study can be found in online repositories. The names of the repository/repositories and accession number(s) can be found in the article/Supplementary Material.
